# Androgen Receptor, Although Not a Specific Marker For, Is a Novel Target to Suppress Glioma Stem Cells as a Therapeutic Strategy for Glioblastoma

**DOI:** 10.3389/fonc.2021.616625

**Published:** 2021-05-21

**Authors:** Nan Zhao, Fei Wang, Shaheen Ahmed, Kan Liu, Chi Zhang, Sahara J. Cathcart, Dominick J. DiMaio, Michael Punsoni, Bingjie Guan, Ping Zhou, Shuo Wang, Surinder K. Batra, Tatiana Bronich, Tom K. Hei, Chi Lin, Chi Zhang

**Affiliations:** ^1^Department of Radiation Oncology, Fred & Pamela Buffett Cancer Center, University of Nebraska Medical Center, Omaha, NE, United States; ^2^Department of Pharmaceutical Sciences, University of Nebraska Medical Center, Omaha, NE, United States; ^3^School of Biological Sciences, University of Nebraska—Lincoln, Lincoln, NE, United States; ^4^Department of Pathology and Microbiology, University of Nebraska Medical Center, Omaha, NE, United States; ^5^Department of Radiation Oncology, Union Hospital of Fujian Medical University, Fuzhou, China; ^6^Department of Radiation Oncology, The First Affiliated Hospital of Hainan Medical University, Haikou, China; ^7^Department of Biochemistry and Molecular Biology, College of Medicine, Eppley Institute for Research in Cancer, University of Nebraska Medical Center, Omaha, NE, United States; ^8^Center for Radiological Research, College of Physicians and Surgeons, Columbia University, New York, NY, United States

**Keywords:** androgen receptor, glioblastoma, cancer stem cells, AR antagonist, enzalutamide

## Abstract

Targeting androgen receptor (AR) has been shown to be promising in treating glioblastoma (GBM) in cell culture and flank implant models but the mechanisms remain unclear. AR antagonists including enzalutamide are available for treating prostate cancer patients in clinic and can pass the blood–brain barrier, thus are potentially good candidates for GBM treatment but have not been tested in GBM orthotopically. Our current studies confirmed that in patients, a majority of GBM tumors overexpress AR in both genders. Enzalutamide inhibited the proliferation of GBM cells both *in vitro* and *in vivo*. Although confocal microscopy demonstrated that AR is expressed but not specifically in glioma cancer stem cells (CSCs) (CD133+), enzalutamide treatment significantly decreased CSC population in cultured monolayer cells and spheroids, suppressed tumor sphere-forming capacity of GBM cells, and downregulated CSC gene expression at mRNA and protein levels in a dose- and time-dependent manner. We have, for the first time, demonstrated that enzalutamide treatment decreased the density of CSCs *in vivo* and improved survival in an orthotopic GBM mouse model. We conclude that AR antagonists potently target glioma CSCs in addition to suppressing the overall proliferation of GBM cells as a mechanism supporting their repurposing for clinical applications treating GBM.

## Introduction

Glioblastoma (GBM) is the most common type of malignant central nervous system tumor in adult patients in the US accounting for about 50% of them (1). Standard treatment for GBM includes maximal safe resection of the tumor, followed by concurrent chemoradiation and adjuvant chemotherapy. Some of the recent promising studies focused on identification of aberrant genetic and signaling pathways to develop small molecules for targeted therapies, characterization of glioblastoma cancer stem cells, modulation of tumor immunological responses and understanding of the rare long-term survivors (2–9). However, even with the extensive efforts of research, current standard care using temozolomide concurrently with brain radiation therapy (RT) after maximal safe surgery only achieves a median survival of fourteen months in the overall patient population, or 22 months in the best prognostic group of patients carrying a hypermethylated MGMT promotor (10, 11). With this universally fatal disease due to its resistance to the standard treatment of RT and chemotherapy, any research advances even small may have a significant impact on survival and provide hope to the thousands of patients who are diagnosed annually with this cancer.

With extensive research conducted to comprehend the molecular regulation of GBM for potential clinical applications, our present knowledge about the tumorigenesis of GBM remains limited. Interestingly, the incidence rate of GBM is significantly higher in adult men than in women as reviewed by Kabat et al. (12). Overall the incidence rate of all glioma in adulthood is also 50% greater in men than in women (13, 14). The exact mechanism underlying this pronounced epidemiology is unclear. Using New York State tumor registry data for the period from 1976 to 1995, McKinley et al. calculated crude, and age- and sex-specific incidence rates for three types of gliomas: glioblastoma, astrocytoma not otherwise specified, and anaplastic astrocytoma (15). Results showed that, overall, males were 1.5 to 2.0 times more likely to develop GBM compared with females even with age justification. In addition, experimental studies indicate that glioblastomas transplanted into animals grow at a slower rate in females compared with males (16). The oncogenic potential of sexual hormones and androgen/androgen receptors cannot be ruled out in the carcinogenesis of GBM. Indeed, steroid hormone receptors including androgen receptor (AR) are members of a superfamily of ligand-activated transcription factors that are potentially oncogenic in gliomas as has been proposed by other researchers (17, 18) and has been confirmed in prostate cancer (19).

In contrast to estrogen receptors (ERs) and progesterone receptors (PRs) whose expressions in human and animal glioma and glioblastoma cell lines are varied and inconsistent (20–26), androgen receptors were consistently detected in a high proportion of gliomas. For example, Caroll et al. investigated the expression of the androgen, estrogen, glucocorticoid, and progesterone receptor messenger ribonucleic acid (mRNA) and protein in a number of astrocytic neoplasms of various histological grades (17). Androgen mRNA was detected in all astrocytic neoplasms examined, regardless of histological subtype. Estrogen receptor mRNA was undetectable in all astrocytic tumors examined in that study. Chung et al. detected AR expression immunohistochemically in 40% of GBMs (grade IV gliomas) and 75% of anaplastic astrocytomas (grade III gliomas) (27). Interestingly, AR expression was also present in 39% of the female glioma samples, similar to the detectable ratio in male (47%). A more recent study from Yu et al. confirmed significantly upregulated AR expression in the GBM tissue as compared to normal peripheral brain tissue in patients by Western blotting assays. Furthermore, AR expression was detected in all eight human GBM cell lines used in this study (28).

AR mediates androgen effects *via* hormone-receptor binding in normal tissues in both male and female although androgen-independent AR activation is a common finding in castration-resistant prostate cancers. Androgens derive predominantly from the testis but also to a lesser extent from the adrenal glands. Testicular testosterone and adrenal (source for females) dehydroepiandrosterone (DHEA) or androstenedione can be converted into bioactive 5α-dihydrotestosterone (DHT) by the enzymes 5 alpha-reductase, which binds to the AR and induces its conformational change. This leads to the dissociation of chaperone and heat shock proteins and the subsequent interaction between AR and co-regulatory molecules and importin α, which facilitates nuclear translocation of AR–ligand complexes. In the nucleus, the AR undergoes phosphorylation and dimerization, which permits chromatin binding to androgen-responsive elements (ARE) within androgen-regulated target genes (29).

To our knowledge, despite these preliminary expression pattern studies of AR in GBM and its known functions/signaling in prostate cancer, there has been no reported studies in confirming the therapeutic role of targeting AR in GBM in brain although a previous study from Zalcman et al. and a very recent report from Werner et al. showed promising results in flank implant models (30, 31). Therefore, we used a syngeneic orthotopic mouse model to test the hypothesis that AR suppression using the AR antagonist, enzalutamide, is effective to suppress tumor growth in the brain. We also studied the expression pattern of AR in GBM tumor specimens from patients treated at our medical center. Simultaneous experiments were conducted in the laboratory on the mechanism of AR inhibition in GBM cell lines in which AR expression status was correlated to the effects of AR inhibition on anchorage-dependent cell growth, tumor sphere formation, as well as cancer stem cell survival/marker gene expression.

## Materials and Methods

### Cell Culture

U138MG (TP53 mutant and PTEN mutant), U87MG (TP53 widetype and PTEN mutant) and Ln229 (TP53 mutant and PTEN widetype) cells were purchased from American Type Culture Collection (ATCC) (Manassas, VA, USA). MGPP-3 (PDGF+, P53−/−, PTEN−/−) which is a murine GBM cell line was kindly provided by Dr. Peter Canoll (Columbia University, New York, NY, U.S.) (32, 33). This cell line was generated from primary cultures from a mouse GBM tumor model induced by injection of VSVG-pseudotyped PDGF-IRES-Cre (PIC) retrovirus, which expresses PDGF and Cre in one transcript, into rostral subcortical white matter (WM) of transgenic mice that carry floxed Pten and p53 (Pten^f/f^; p53^f/f^), and stop-floxed luciferase reporter. The human GBM cells were cultured in DMEM (Thermo Fisher Scientific, Inc., Waltham, MA, USA) or EMEM (ATCC, Manassas, VA, USA) per provider’s recommendation supplemented with 10% fetal bovine serum (FBS) (Thermo Fisher Scientific, Inc., Waltham, MA, USA), 100 U/ml penicillin and 100 mg/ml streptomycin in a humidified incubator with 5% CO_2_ at 37°C. Murine GBM cells were cultured with previously published protocol (32, 33).

### Cell Proliferation Assay

Cell titer blue assays were performed with cells cultured in 96-well plates treated with different concentrations of AR antagonists (enzalutamide and bicalutamide (Selleckchem, Munich, Germany) for 48 h before changing to fresh media and continuing culture overnight. 20 µl cell titer blue reagents (Promega, Madison, WI, USA) were added to each well containing 100 µl medium. After incubation at 37°C for 2 h, the fluorescence was read at 560/590 nm using SpectraMax (Molecular Devices, San Jose, CA, USA). IC50s of AR antagonists on GBM cell lines were calculated using the GraphPad software (Version 8.3.1, San Diego, CA, USA).

### Confocal Immunofluorescence Microscopsy

U87MG, U138MG, and MGPP3 GBM cell lines were treated with DMSO (control), 20 µM, or 40 µM enzalutamide for 48 h. Cells were fixed with 4% paraformaldehyde for 10 min at room temperature, permeabilized with 0.5% Triton X-100 for 10 min and then washed in PBST three times. Cells were blocked with 1% BSA for 30 min and then incubated with the primary antibodies for 1 h at room temperature. The primary antibodies include anti-c-Myc (1:100) (Abcam, Cambridge, MA, USA) and AR antibody (441) (1:50) (Santa Cruz Biotechnology, Inc., Dallas, TX, USA). After incubating with primary antibodies, cells were washed with PBST three times, 5 min each and then incubated with secondary antibody conjugated with Alexa Fluor 488 or Alexa Fluor 647 (Abcam, Cambridge, MA, USA) for 1 h at room temperature. Cell nuclei were stained with DAPI mounting medium (Thermo Fisher Scientific Inc., Waltham, MA, USA) before captured with the LSM800 confocal microscope (ZEISS, Germany). Similar procedures were performed for FFPE mouse brain tumor specimens for confocal microscopy with the following primary antibodies used: anti-AR antibody (ab3510) (Abcam, Cambridge, MA, USA), anti-Nanog (PA5-85110) (Thermo Fisher Scientific Inc., Waltham, MA, USA), and anti-CD133 antibody (ab19898) (Abcam, Cambridge, MA, USA). The weighted colocalization was analyzed for 4 different areas of the confocal images using ZEN colocalization software (ZEISS, Germany).

### Tumor Spheroid Formation and Treatment

U87MG and MGPP3 cells were cultured in media with 0.5% FBS in 96-well plates (ultra-low attachment) (Corning, Inc., Corning, NY, USA) at a density of 10,000/well and maintained at 37°C under 5% CO_2_ in a humidified incubator. After tumor spheroids were formed, DMSO (control), enzalutamide, or bicalutamide at specified concentrations were added into culture media. The diameters of the spheroids were monitored every day for an additional 3–4 days under a microscope and growth curves of the spheroids were plotted and compared between groups.

### Flow Cytometry on Cancer Stem Cells in Tumor Spheroids/Cell Culture

The subpopulation of cancer stem cells in GBM tumor spheroids or cultured adherently were sorted and evaluated with and without enzalutamide treatment, respectively, with an anti-CD133 CSC surface antibody (Miltenyi Biotec, Germany). After treating the tumor spheroids for 4 days with 120 µM enzalutamide or 180 µM bicalutamide, the tumor spheroids were harvested, gently dissociated to single cell suspensions using ACCUTASE™ (STEMCELL Technologies Inc., Canada). First a total of 10^6^ cells were stained with Live/Dead fixable dead cell staining dyes and then incubated with APC-conjugated anti-CD133 antibody (Miltenyi Biotec, Germany). After 30 min of incubation with CD133 antibody at 4°C, cells were washed with phosphate-buffered saline (PBS) at 300×*g* for 10 min. Samples were sorted using a FACS LSRII G Flow Cytometer and percentages of CSC subpopulation were analyzed by FACSDiva software (Beckon-Dickinson, Franklin Lakes, NJ, USA).

### Limiting Dilution Assays *In Vitro* and *In Vivo* on Stem Cell Content

To determine the content of CSCs or stem-like cells in the cultured GBM cell lines with or without enzalutamide treatment, U87MG or MGPP3 cells were treated with DMSO (control) and enzalutamide for 2 days in adherent cultures before being trypsinized and dissociated into suspended single cells. A series of numbers of suspended single cells were seeded in ultra-low attachment 96-well plates (Corning, Inc., Corning, NY, USA) at 1, 10, 25, 50 and 100 cells/well and cultured in media with 0.5% FBS for 14 days with an intermittent assessment to confirm the formation of tumor spheroids. After 14 days, the numbers of wells that have at least one sphere were counted manually under a microscope.

To further confirm the change of CSCs with or without enzalutamide treatment, orthotopic LDA *in vivo* experiment was performed. MGPP3 cells were treated with DMSO (control) or enzalutamide for 3 days before dissociating into single cells. 10^3^ and 10^4^ cells with or without enzalutamide treatment were inoculated into six mice brain for each group. The growth of tumor was monitored using PerkinElmer *In vivo* Imaging System (IVIS) every week.

The frequency of CSCs or stem-like cells was calculated using an Extreme Limiting Dilution Algorithm (ELDA software;http://bioinf.wehi.edu.au/software/elda/).

### Western Blotting

After treating U87MG spheroids with 120 µM enzalutamide for 1 day or 3 days, the cell pellets were homogenized with RIPA Lysis and Extraction Buffer (Thermo Fisher Scientific Inc., Waltham, MA, USA) with a mixture of protease inhibitors (Thermo Fisher Scientific Inc.). The protein concentrations were determined using NanoDrop 2000 spectrophotometer (Thermo Fisher Scientific Inc.). Cell lysates containing 100 µg protein were electrophorized on 4–20% Tris-Glycine SDS-PAGE gel (Thermo Fisher Scientific Inc.). The primary antibodies used in this experiment include polyclonal antibody Nanog (PA5-85110, 1:1,000) (Thermo Fisher Scientific Inc.), monoclonal anti-Oct4 antibody (ab181557, 1:500) (Abcam, Cambridge, MA, USA), polyclonal anti-AR antibody (ab3510, 1:500) (Abcam, Cambridge, MA, USA), monoclonal anti-c-Myc (ab32072, 1:1,000) (Abcam, Cambridge, MA, USA), monoclonal ani-CDC25A (DCS-120) (MA5-13794, 1:1,000) (Thermo Fisher Scientific Inc.), polyclonal anti-GADD45A (ab180768, 1:1,000) (Abcam, Cambridge, MA, USA), polyclonal anti-FOXO3a (ab70315, 1:1,000) (Abcam, Cambridge, MA, USA), polyclonal anti-GATA4 (PA1-102, 1:1,000) and monoclonal anti-β-actin (AC-74, 1:3,000) (MilliporeSigma, Burlington, MA, USA). All these antibodies have cross-reactivity against both human and murine antigens. After incubating the blots with the primary antibody overnight at 4°C, the blots were washed three times for 5 min with TBST and then incubated with the IRDye 800CW Goat Anti-Rabbit LgG (H+L) or IRDye 800CW Goat Anti-Mouse LgG (H+L) secondary antibodies (Li-Cor Biotechnology, Lincoln, NE, USA) for 1 hour at room temperature. The blots were scanned with densitometry performed using Odyssey XL imager and provided software (Li-Cor Biotechnology, Lincoln, NE, USA)

### CSC Marker Gene Expression Analysis With TCGA Database

Spearman’s rank correlation coefficients of mRNA expression levels between AR and various CSC marker genes were calculated based on RNA-seq results of GBM patients from TCGA database.

### RNA-seq and Quantitative Real-Time PCR

U87MG cells were treated with 80 µM enzalutamide for 4, 24, and 48 h before the total RNA was isolated using RNeasy Plus Mini Kit (Qiagen, Netherlands). The RNA-seq was performed by next-generation sequencing (NGS) using NextSeq550 (Illumina, San Diego, CA, USA). For data analyses, each RNA-seq read was trimmed using Trimmomatic (34) to make sure the average quality score is larger than 30 and the minimum length being 30 bp or longer. Reads were mapped to the human genome (NCBI build 37) using Tophat v2.1.1 (35), which together accurately aligned an average of 90% of paired-end reads. Numbers of reads in genes were counted by the software tool of HTSeq-count (36) using corresponding human gene annotations and the “union” resolution mode was used. Differential expressions were computed for whole gene regions by summing reads for each region. For pair-wise differential expression comparisons, DESeq (v.1.36.0) (37) was used to analyze the numbers of reads aligned to genes and to identify differentially expressed genes. A threshold value for fold-change of differential expression was set at log2 (fold-change) >1 (two-fold actual value) and adjusted P-values <0.05 for rejecting the null hypothesis.

Quantitative real time PCR (qPCR) was performed using Taqman probes from Applied Biosystems (Thermo Fisher Scientific Inc.) on the reversely transcribed cDNAs from the same RNA samples used in RNA-seq to confirm the changes of the genes with or without enzalutamide treatment. Fold changes of the gene expression levels were calculated by the delta Ct method relative to the control samples. The beta-actin was used as internal control for normalization.

The primer sequences used are as follows:

AR-F: ACCGAGGAGCTTTCCAGAATC,
AR-R: AGGCTCTGGGACGCAACCT;
Sox2-F: CACACTGCCCCTCTCAC,
Sox-R: TCCATGCTGTTTCTTACTCTCC;
OCT4-F: TCTCCCATGCATTCAAACTGAG,
OCT4-R: CCTTTGTGTTCCCAATTCCTTC;
NANOG-F: GAAATACCTCAGCCTCCAGC,
NANOG-R: GCGTCACACCATTGCTATTC;
CD133-F: GTGTCCTGGGGCTGCTGTTTA,
CD133-R: CCATTTTCCTTCTGTCGCTGG;
Beta-actin-F: AGAAAATCTGGCACCACACC,
Beta-actin-R: AGAGGCGTACAGGGATAGCA

### Syngeneic Orthotopic GBM Mouse Model

5 × 10^4^ MGPP3 murine glioblastoma cells were stereotactically implanted into the right brain hemisphere of 16 to 17 week-old male mice weighing 20 to 30 g. The growth of the tumor was monitored using PerkinElmer *In vivo* Imaging System (IVIS) each week. Mice were imaged 10 min after intraperitoneal injection of luciferin (Biosynth International, Inc., Itasca, IL, USA) at 150 mg/kg. The mice were regrouped into vehicle (negative control) (10% DMSO, 30% PEG400, 60% corn oil) or enzalutamide treatment groups with equivalent mean values of bioluminescence signals between groups at week 5 after the implantation. Enzalutamide (20 mg/kg, dissolved in the vehicle solution, 100 µl/injection) or vehicle only (100 µl/injection) were injected intraperitoneally (IP) into the mice three times per week per previously published protocols (38). The treatment was continually given to the mice until week 15 after the implantation (week 10 after starting drug treatment) or death. The mice presented with signs of near death such as seizures were euthanized with neck dislocation. After death was confirmed, mice were perfused with 10% formalin in PBS and brain tissues were dissected for immunohistochemistry (IHC) studies. All studies were carried out in compliance with the local ethical guidelines for animal experiments. The protocol was approved by the Institutional Animal Care and Use Committee (IACUC) of University of Nebraska Medical Center (protocol #: 16-134-01). All the mice with tumor received palliative care for pain control after surgery, during the follow-up and prior to euthanization per institutional guidelines.

### Immunohistochemistry

Serial unstained slides were cut from the formalin-fixed paraffin-embedded (FFPE) tissue blocks of GBM specimens from deceased, normal brain autopsy tissue from patients who died of non-neurological disease, and temporal lobectomy surgical specimens from patients with epilepsy with approved protocol from our Institutional Review Board. IHC for AR (clone SP107 rabbit monoclonal antibody, Cell Marque, Rocklin, CA, USA) was performed using BenchMark Ultra IHC/ISH system (Roche, Basel, Switzerland). Slides cut from the FFPE mouse brain GBM tissue were incubated with anti-AR (ab3510), anti-CD133 (ab19898), anti-Sox2 (ab97959) and anti-c-Myc (ab32072) individually or in combination. All these antibodies were from Abcam, Cambridge, MA, USA. After staining for the above markers with substrates incubated and color developed, slides were scanned with Ventana iScan HT slide scanner at 400× magnification and quantified using Definiens Tissue Studio (Ventana, Munich, Germany).

### Statistics

Experimental data for cell proliferation assays, tumor spheroid sizes and IHC signals were calculated as Mean ± standard error of the mean. Student t-test (two groups) or one-way ANOVA (more than two groups) was performed using GraphPad Software (Version 8.3.1, San Diego, CA, USA). Overall survivals (OS) were compared between enzalutamide treatment and vehicle only groups of mice with Kaplan–Meier analysis. Results were considered statistically significant if p <0.05.

## Results

### AR Is Commonly Overexpressed in GBM Tumor Specimen From Patients

**Figure 1:**
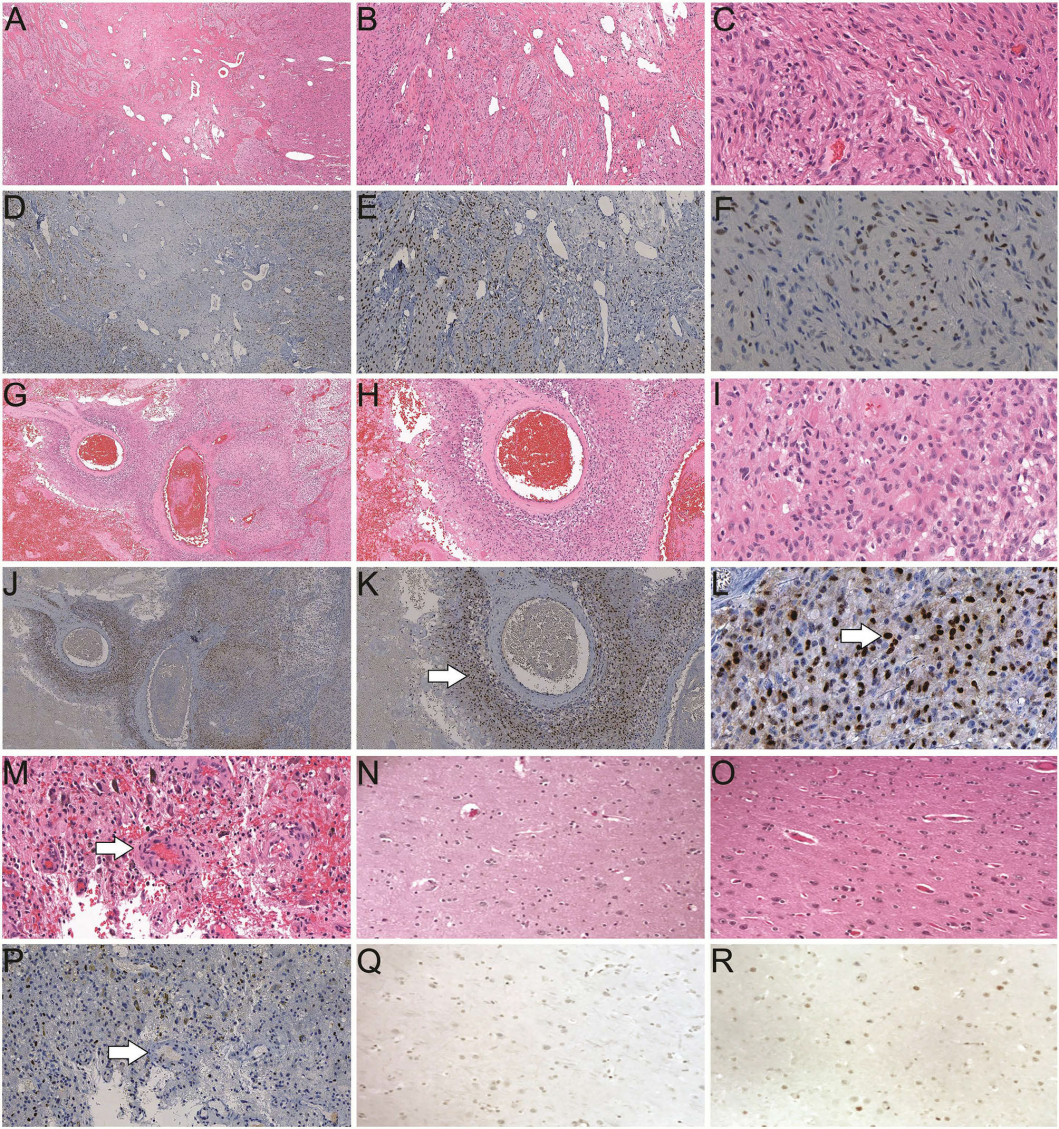
Histological studies of androgen receptor (AR) expression in human brain/GBM tissue. **(A–C)** H&E staining of a GBM slide from a male patient (40×, 100× and 400× magnification, respectively). **(D–F)** Positive AR expression (brown) in a GBM slide from the same patient as A (40×, 100× and 400× magnification, respectively). **(G–I)** H&E staining of a GBM slide from a female patient (40×, 100× and 400× magnification, respectively). **(J–L)** AR staining in a GBM slide from the same patient as G (40×, 100× and 400× magnification, respectively). Enriched AR positive cells at peri-vascular area are best shown in **K** (arrow). Majority of AR staining (brown) is in nuclei in a subset of the cells (arrow in **L**). **(M)** H&E staining of a GBM specimen showing the endothelial proliferation of the vessels (arrow). **(P)** AR staining of the GBM specimen from the same patient as M. **(N)** H&E staining of a normal human brain autopsy specimen. **(Q)** Negative AR staining of the normal brain autopsy specimen from the same patient as N, at 400× magnification. **(O)** H&E staining and **(R)** scattered weakly positive nuclear staining of AR in a temporal lobectomy surgical specimen from a patient with epilepsy, at 400× magnification.

We performed immunohistochemistry (IHC) studies on tumor specimens from GBM patients and demonstrated overexpression of AR in tumor tissues when compared with control brain specimens (brain tissue from patients without neurologic disease/tumor or patients with temporal lobectomy for epilepsy as shown in **Figure 1**). The majority of both male and female GBMs were found to have high AR nuclear expression levels in a significant percentage of cells in tumor (**Figures 1F, L**). We observed the pattern of peri-arterially enriched AR expression (**Figure 1K**). In normal brain tissue controls from autopsy, no AR expression was detected (**Figure 1Q**). Similarly, lobectomy tissue from epilepsy patients showed very low AR expression detected in very few cells (**Figure 1R**). Forty-three out of 58 GBM patients (74%) examined so far in our database display positive AR expression in >10% tumor cell nuclei, with the other eleven patients showing 1–10% positivity (93% with >1% positivity) and only four patients’ tumor were found to be completely devoid of AR staining. Ninety-seven percent male and 87.5% female patients, respectively, were found to be positively stained for AR in >1% of tumor cell nuclei (P = 0.33). Seventy-nine percent male and 66.7% female patients showed positive AR staining in >10% tumor cell nuclei (P = 0.30). Reviewing of the staining pattern and positivity counting were performed independently by two pathologists from our institute with a high level of consistency.

### AR Antagonists Inhibit the Proliferation of GBM Cells *In Vitro*

**Figure 2:**
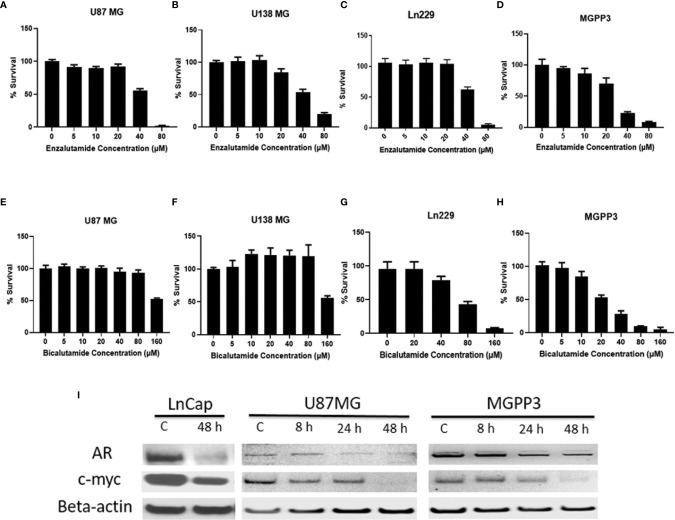
AR antagonists inhibit proliferation of human and mouse GBM cell lines *in vitro*. **(A–D)** GBM cell lines were treated with indicated concentrations of enzalutamide for 2 days before cell titer blue assays were performed. **(A)** U87MG cell line (human) (IC50 = 41 μM). **(B)** U138MG cell line (human) (IC50 = 45 μM); **(C)** Ln229 cell line (human) (IC50 = 41 μM). **(D)** MGPP3 cell line (mouse) (IC50 = 56 μM); **(E–H)** GBM cell lines were treated with indicated concentrations of bicalutamide for 2 days before cell titer blue assays. **(E)** U87MG cell line (IC50 = 121 μM). **(F)** U138MG cell line (IC50 = 122 μM). **(G)** Ln229 cell line (IC50 = 72 μM). **(H)** MGPP3 cell line (IC50 = 42 μM). **(I)** Western blotting assays demonstrate that AR and c-Myc protein levels decrease in the U87MG, MGPP3 and LnCap cells (a prostate cancer cell line for positive control) after the treatment of enzalutamide (40 µM) time dependently. 8, 24, 48 h: cells were treated with the drug for 8, 24 or 48 h. All experiments were performed with three independent replicates.

We demonstrated that in commercially available GBM cell lines including A172, Ln229, M059K, U87MG and U138MG, all are AR-positive but with variable expression levels (**Figure 1S**). Our results are consistent with the finding from Yu et al. who showed all twelve GBM cell lines tested were expressing AR (28). AR antagonists, enzalutamide or bicalutamide, inhibited the proliferation of GBM cells and significantly reduced viability after two days of treatment in a dose-dependent manner in all human and murine GBM cell lines tested *in vitro* (**Figure 2**). The GBM cells’ sensitivity to the drug was not found to be related to the level of AR expression. Even though AR expression levels are relatively low in some cell lines such as U87MG and Ln229, they were still susceptible to AR antagonists with IC50s of enzalutamide and bicalutamide being ~40 and 80–160 µM, respectively, for all tested human GBM cell lines.

### Enzalutamide Downregulates c-Myc and AR Expression in a Dose-Dependent Manner

**Figure 3:**
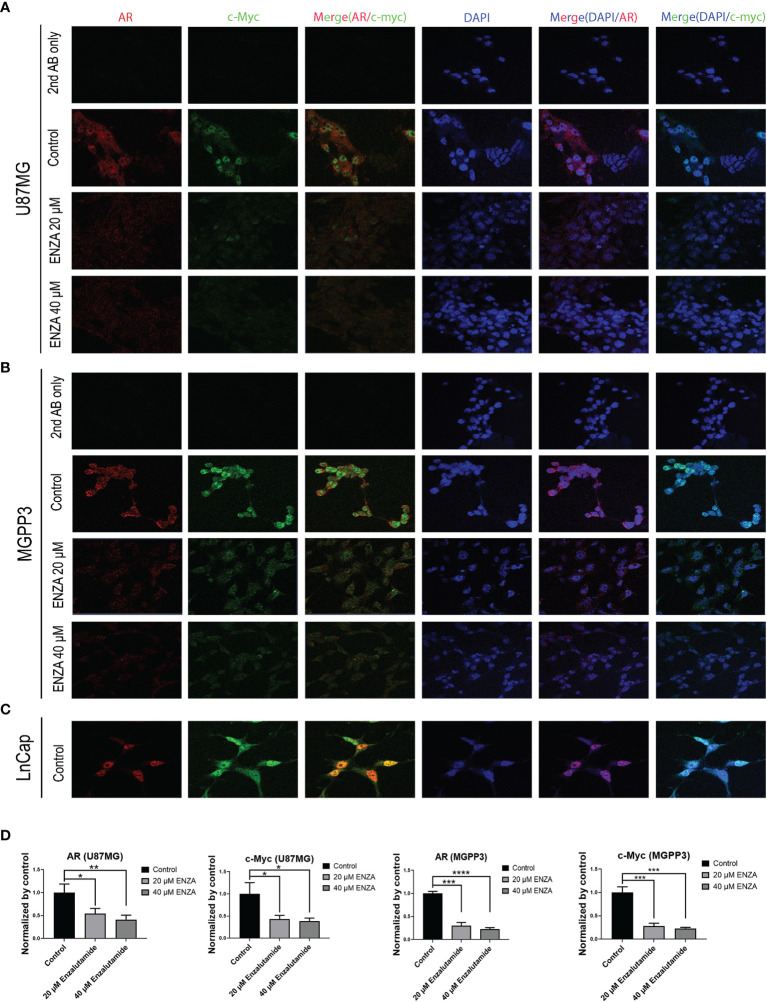
Confocal immunofluorescence studies demonstrate that enzalutamide decreases AR (red) and c-Myc (green) in GBM cells cultured *in vitro* (U87MG and MGPP3). **(A)** U87MG cells were treated with DMSO (negative control) or various concentrations of enzalutamide (20 µM or 40 µM). **(B)** MGPP3 cells were treated with DMSO or various concentrations of enzalutamide (20 µM or 40 µM). **(C)** LnCap (prostate cancer cell line) served as a positive control. **(D)** Quantification of the immunofluorescence signals of AR and c-Myc in U87MG and MGPP3 cell lines after enzalutamide treatment (20 µM or 40 µM) for 24 h. *p < 0.05; **p < 0.01; ***p < 0.001; ****p < 0.0001.

c-Myc, an extensively studied oncogene, has an important role in ensuring tumor development, promoting proliferation and maintenance of cancer progenitor cells in human cancers (39–41). c-Myc, along with other stem cell genes including SOX2, BMI1 and OCT-4, is highly expressed in prostate cancer stem/progenitor cells (42). We studied the relationship between c-Myc expression and AR blockade in GBM cells. Human prostate cancer cell line LnCap, an AR-positive cell line, was used as a positive control for AR and c-Myc expression in GBM cells (**Figure 3A**). Both AR and c-Myc expression levels in U87MG and MGPP3 cell lines were downregulated after 20 µM enzalutamide treatment for 24 h, and both decreased further with higher concentration of enzalutamide (40 µM) (**Figures 3B, D**). The downstream genes of c-Myc such as FOXO3a and CDC25A also decreased significantly or with strong trends at the protein level after enzalutamide treatment in both U87MG and MGPP3 cells. Another downstream gene GADD45A showed significant decrease in MGPP3 cells but not in U87MG after drug treatment (**Figure 2S**). MGPP3 murine GBM cells showed similar AR expression patterns and dose-dependent response to enzalutamide treatment (**Figures 3C, D**). However, unlike LnCap prostate cancer cells with nuclear specific AR distribution, both GBM cell lines cultured *in vitro* showed cytosol-enriched subcellular localization of AR which is in contrast with the nuclear dominant localization of GBM patients’ specimens based on IHC staining.

### AR Antagonists Suppress the Growth of Spheroids and Decrease Glioma Cancer Stem Cell Population in U87MG Cells *In Vitro*

Cancer stem cells (CSCs) can be enriched in spheroids using an ultra-low concentration of serum (43). Although the consensus has not been reached on what is/are the most representative marker(s) to detect CSCs in GBM, cell surface marker CD133 has been the most commonly used in used (44, 45).

**Figure 4:**
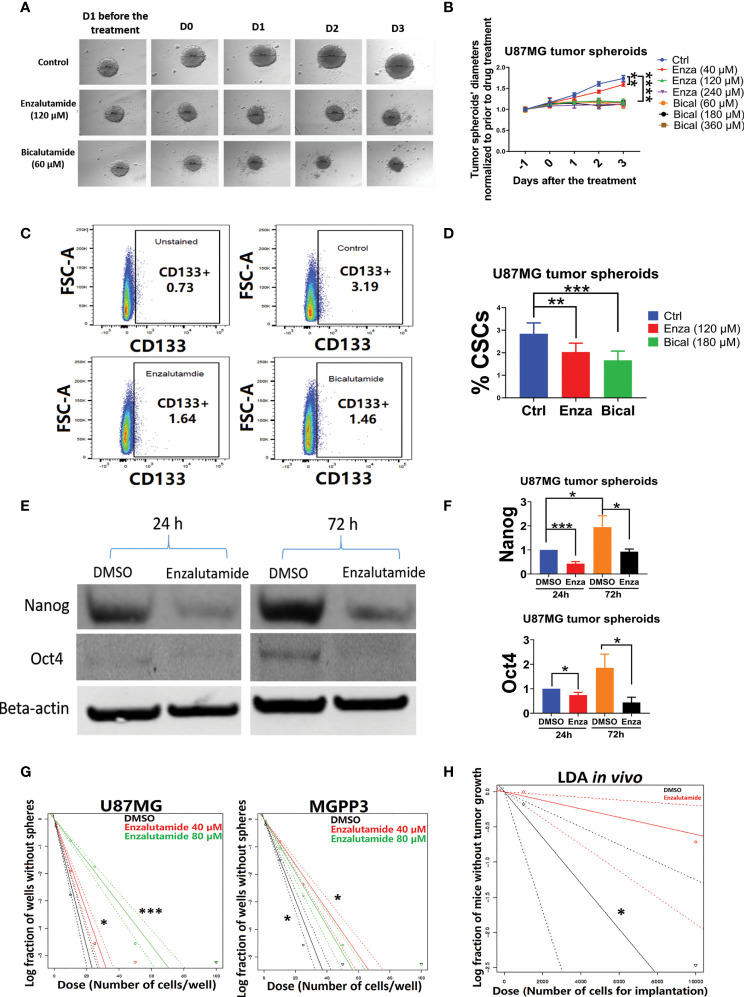
AR antagonists decrease the cancer stem cell population in U87MG GBM cells *in vitro*. U87MG spheroids were cultured in low serum medium (0.5%) for 2 days and then treated with DMSO (negative control), enzalutamide (120 µM), or bicalutamide (180 µM) for another 4 days with diameters of spheroids measured daily. **(A)** Representative figures of the U87MG spheroids before and after the treatment of enzalutamide (120 µM) or bicalutamide (60 µM). **(B)** Measured diameters of the spheroids normalized to before the treatment during drug treatment with increasing concentrations of enzalutamide (40, 120, and 240 µM) or bicalutamide (60, 180, and 360 µM). **(C)** Representative flow cytometry gating strategy with dissociated cells from U87MG spheroids. Upper left: no drug treatment and no CD133 antibody staining; upper right: DMSO only (no drug treatment control); lower left: enzalutamide (120 µM); lower right: bicalutamide (180 µM). **(D)** CSC populations (CD133+) were compared in dissociated cells from spheroids with or without treatment of AR antagonists using flow cytometry as shown in **(C)** Eight replicates per group were used for flow cytometry with mean values presented in the histogram. **(E)** Western blotting assays showed decreased expression levels of CSC markers, Nanog and Oct4, after enzalutamide treatment for 24 and 72 h. **(F)** Quantification of Nanog and Oct4 protein levels before and after the treatment of enzalutamide for 24 and 72 h. All experiments were performed with three independent replicates. **(G)** Extreme Limiting Dilution Assays (ELDA) *in vitro* to estimate the frequency of tumor sphere-forming stem-like cells in U87MG and MGPP3 cell lines. Plot of the log (e) fraction of wells without tumor spheres as a function of plated U87MG/MGPP3 cell number/well. The more vertical the line, the higher the percentage of tumor sphere-forming cells or CSCs. **(H)** Extreme Limiting Dilution Assays (ELDA) *in vivo* to estimate the frequency of CSCs. Plot of the log (e) fraction of mice without tumor growth as a function of implanted MGPP3 cells. *p < 0.05; **p < 0.01; ***p < 0.001; ****p < 0.0001.

After the formation of spheroids of U87MG in culture media with an ultra-low concentration of serum, incubation of the spheroids with AR antagonists suppressed their further growth (**Figures 4A, B**). In contrast, untreated (DMSO controls) spheroids continued growing in culture media but the growth was delayed or completely arrested with increasing concentrations of AR antagonists added. After treatment with either enzalutamide or bicalutamide, CD133+ cells in U87MG spheroids were significantly decrease proportionally compared with the control group treated with DMSO only based on flow cytometry (**Figures 4C, D**). The average percentages of CSC cells in spheroids were 3.1 ± 0.3, 2.2 ± 0.1 and 1.6 ± 0.2 in DSMO control, enzalutamide and bicalutamide, respectively.

### AR Antagonists Downregulate Cancer Stem Cell Marker Gene Expression in GBM Cells *In Vitro* in a Time-Dependent Manner

In addition to the cell surface marker CD133, other cancer stem cell markers/embryonic stem cell markers such as Nanog and Oct4 have also been widely used for cell linage studies in cancers including GBM (46–50). The expression levels of Oct4 and Nanog in the spheroids of U87MG with and without enzalutamide treatment were studied with Western blotting experiment. We observed enrichment in stemness markers Nanog and Oct4 in U87MG cells over 72 h of culturing time in spheroids when treated with solvent control only (**Figure 4E**). Enzalutamide treatment significantly decreased expression of both Nanog and Oct4 proteins in spheroids after only one-day incubation with the drug as compared to controls with DMSO treatment only. The proportional reduction, relatively to DMSO treatment control, of Nanog and Oct4 became more significant after prolonged treatment (three days) of enzalutamide (**Figure 4F**). GATA4, a downstream gene of Oct4 and Nanog, also decreased significantly in its protein expression after the treatment of enzalutamide (**Figure 2S**).

### *In Vitro* and *In Vivo* Limiting Dilution Assays Demonstrate That Enzalutamide Suppresses Tumor Sphere-Forming Capability in GBM Cells *In Vitro* and Tumor Formation *In Vivo*

The *in vitro* limiting dilution assay (LDA) has been used widely to analyze the cancer stem cell population under various culturing conditions (51–54). Extreme limiting dilution assay (ELDA) is a software application that calculates the proportion of cancer stem cells in a mixed cell population with statistical software (55). Our ELDA experiments demonstrated that enzalutamide treatment significantly decreased the tumor sphere-forming capacities from the subpopulation of cancer stem cells in both U87MG and MGPP3 cell lines (**Figure 4G**). The inhibitory effects of the AR antagonist are dose-dependent with 80 µM enzalutamide exhibiting a significantly enhanced effects suppressing tumor sphere formation/CSC subpopulation in U87MG cell line compared with the negative control or lower concentration of enzalutamide (40 µM). Interestingly, the inhibitory effects of enzalutamide on tumor sphere formation in MGPP3 cells are also significant compared to the negative control but seem to be saturated with doses above 40 µM (**Figure 4G**).

LDA *in vivo is* the gold standard to test the tumor-initiating capability of the CSCs. With each mouse brain inoculated with 10^4^ MGPP3 cells, all six mice in the control group without drug pretreatment had tumor growth as expected, whereas only three out of six in the enzalutamide pretreated group had tumor growth after 6 weeks. In mice inoculated with decreased number of tumor cells at 10^3^ cells each, one out of six mice in the control group had tumor growth while zero out of six mice in the enzalutamide pretreated group had tumor growth 6 weeks after implant. ELDA software estimated that the ratios of CSCs with tumor-forming capacity in the cell line decreased from 1/3,071 to 1/16,498 after the treatment of enzalutamide (p = 0.022) (**Figure 4H**).

### mRNA Expression Levels of AR Are Positively Correlated With GBM CSC Marker Genes as Well as Genes/Pathways Related to Proliferation

**Figure 5:**
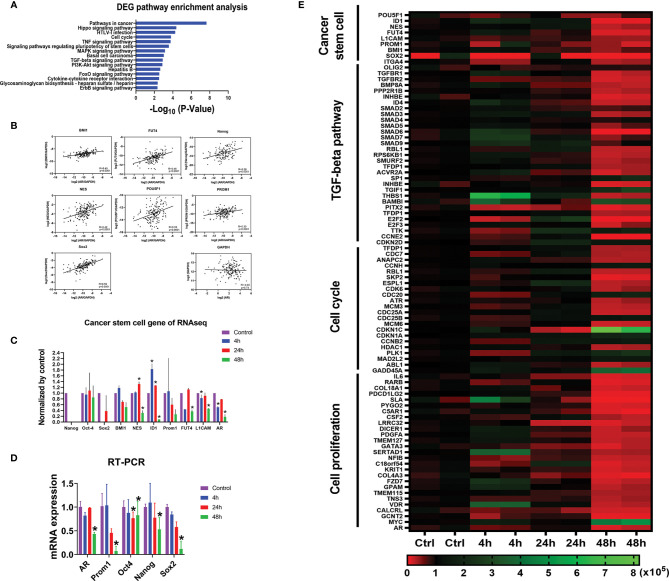
AR positively correlates with CSC marker genes at mRNA expression level. **(A)** Gene Set Enrichment Analyses (GSEA) against the KEGG pathway database showing the top 15 pathways most significantly regulated in the U87MG cells with differentially expressed genes (DEGs) after the treatment of enzalutamide for 48 h. **(B)** The positive correlations of mRNA expressions between CSC marker genes and AR from *in vivo* RNA-seq results in GBM patients from TCGA database. GAPDH, a housekeeping gene, showed no correlation with AR (R = -0.03). **(C)** mRNA expression levels of various CSC marker genes in U87MG cells cultured *in vitro* before and after the treatment of enzalutamide (80 µM) from RNA-seq results from our laboratory. CD133 is an alias name for Prom1. **(D)** Quantitative RT-PCR results of the representative CSC genes after the treatment of enzalutamide. **(E)** Heatmap of the genes specific for cancer stem cell, TGF-β signaling pathway, cell cycle, and cell proliferation responding to enzalutamide treatment in U87MG cells at different time points. The scale bar at the bottom represents the normalized read counts of the genes from RNA-seq results. *p < 0.05.

To explore the correlation between AR gene and GBM cancer stem cell genes in The Cancer Genome Atlas (TCGA) database, we selected 10 well-known GBM cancer stem cell genes (45, 56). We found that the mRNA expression levels of all GBM cancer stem cell genes are positively correlated with the AR gene, the highest correlation being SOX2 (Spearman’s rank correlation coefficient R = 0.59) (**Figure 5B**). No correlation was seen between the expression levels of AR and GAPDH, a housekeeping gene.

In addition, from our RNA-seq analyses on U87MG cells cultured *in vitro*, we found that almost all cancer stem cell marker genes such as Nestin, ID1, FUT4 and L1CAM showed either a significantly decreased or trends of decreased expression (Nanog, CD133 (Prom1), Sox2 and BMI1) after enzalutamide treatment (80 µM) for 48 h. Quantitative RT-PCR assays were further performed and confirmed that the mRNA expression levels of AR, CD133 (Prom1), Oct4, Nanog, and Sox2 were all significantly decreased after the treatment of enzalutamide (80 µM) for 48 h (**Figure 5D**).

Based on the RNA-seq results after treating U87MG cells with enzalutamide for 48 h, comprehensive Gene Set Enrichment Analyses (GSEA) analyses against the KEGG pathway database (https://www.genome.jp/kegg/pathway.html [genome.jp]) were also performed to identify additional cellular functions of AR. Cancer stem cell signatures were included in different related pathways. The signaling pathways regulating pluripotency of stem cells specifically FoxO and TGF-β signaling pathways, as expected, are listed among the top 15 pathways most affected after enzalutamide treatment, with 32 differentially expressed genes (DEGs) after the treatment of enzalutamide were enriched in the “hsa04550: Signaling pathways regulating pluripotency of stem cells” (p = 2.72 × 10^−4^). In addition, genes/pathways involved in cell cycle, Hippo, MAPK, PI3K-Akt and ErbB signaling pathways are listed as well indicating the involvement of AR in promoting cell cycling/proliferation of differentiated GBM cells (**Figure 5A**). Furthermore, the heatmap generated from RNA-seq results confirmed the downregulation of the expression levels of not only the genes specific for cancer stem cells but also those in TGF-β signaling, cell cycles and cell proliferation in U87MG cells after enzalutamide treatment time-dependently (**Figure 5E**).

### Enzalutamide Downregulates Cancer Stem Cell Marker Gene Expression *In Vivo*, Inhibits GBM Tumor Progression and Significantly Prolongs Survival in Mice

**Figure 6:**
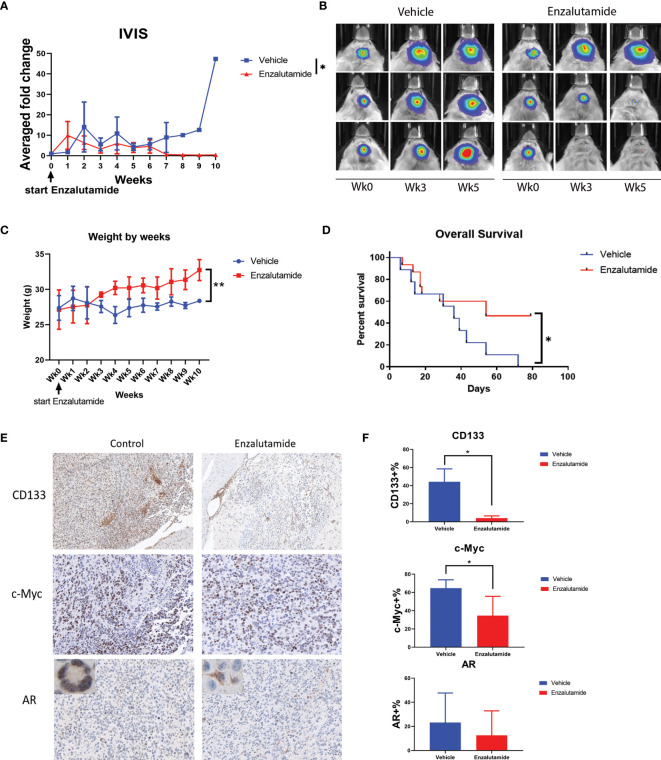
Enzalutamide suppresses CSC marker gene expression, GBM tumor growth *in vivo* and prolongs overall survival in treated mice with MGPP3 cells implanted in brain. **(A)** Fold changes of the bioluminescent signals of the tumors in brain after the treatment of vehicle only or enzalutamide (20 mg/kg, three times per week, IP). **(B)** Representative IVIS images of the tumor growth in mouse brain after vehicle only or enzalutamide treatment. Wk 0, 3 and 5: IVIS imaging taken prior to (week 0), 3 and 5 weeks after initiating of drug injection. Mann–Whitney *U* tests were performed in both **(A, B)** to compare between groups. **(C)** Weight changes of the mice after the treatment. **(D)** Overall survival was significantly improved in mice treated with enzalutamide comparing with vehicle only. **(E)** Representative images of the mouse brain tissue after IHC staining for CD133 (100×), c-Myc (200×), and AR (200×). Corner image (left upper corner of the left panel): high magnification image showing tumor cells with positive AR staining with nuclear localization without enzalutamide treatment. Corner image (left upper corner of the right panel): high magnification image showing tumor cells with positive AR staining but more cytosol distribution after enzalutamide treatment. **(F)** Quantifications of positive cells in the brain tumors from IHC staining after vehicle only or enzalutamide treatment. Two-tailed student t tests were performed for statistical comparisons. *p < 0.05; **p < 0.01.

**Figure 7:**
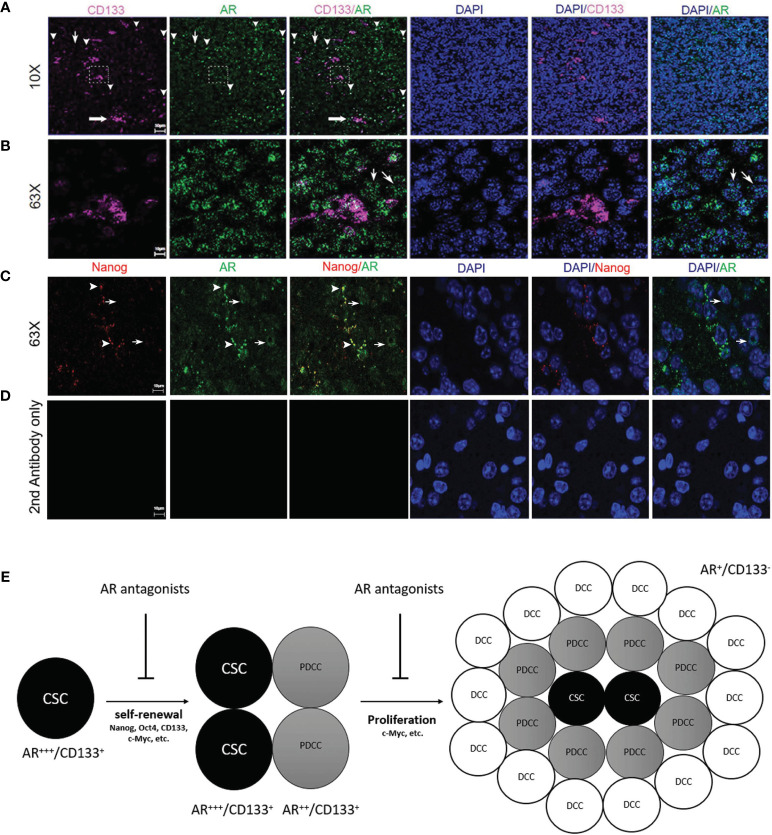
Immunofluorescence confocal microscopy on mouse GBM FFPE specimen (5 µm thickness) showing the subcellular localizations of AR (green), CD133 (pink) and DAPI-stained nuclei (blue). **(A)** Low magnification images (10×) and **(B)** high magnification images (63×) which were scanned from areas in the dash line boxes in **(A)** demonstrated co-expression of CD133 in AR-expressing (AR+) cells with high staining intensity (arrowheads). AR expression can be seen in CD133 negative cells but with low AR staining intensity (thin arrows). **(C)** High magnification images (63×) demonstrated co-expression of Nanog in AR+ cells with high AR staining intensity (arrowheads). Cells with negative Nanog staining could be AR+ but showed weaker AR staining intensities than Nanog+ cells (thin arrows). **(D)** High magnification images (63×) with slides stained with secondary antibodies only as negative controls. **(E)** Schematic figure showing the proposed mechanisms of AR antagonists inhibiting both cancer stem cells maintenance and cancer proliferation from more differentiated cells. CSC, cancer stem cell; PDCC, partially differentiated cancer cell; DCC, differentiated cancer cell.

We further examined the effects of the AR antagonist using a syngeneic orthotopic GBM mouse model. MGPP3 cells which express luciferase constitutively were intracranially injected into mice, and treatments (enzalutamide vs. vehicle control) were administered twice per week once tumors developed. Bioluminescence imaging was used to monitor the differences in tumor progression between treatment groups (**Figures 6A, B**). Representative IVIS images of progressed tumors (top right) and tumors responded to enzalutamide treatment (two mice at bottom right) are shown in **Figure 7B**. We found that GBM tumor growth was suppressed (size reduced or stabilized) in five out of nine mice (55.6%) in the enzalutamide-treated group *versus* zero out of nine mice (0%) in the vehicle only control group. Mice tolerated this dose of enzalutamide treatment well with significantly more weight gain during the course of treatment (**Figure 6C**).

Furthermore, mice treated with enzalutamide had significantly improved overall survival compared with those in the control group (p <0.05). The median survival for the control and enzalutamide-treated groups was 36 days and 54 days, respectively (**Figure 6D**). All the mice in the control group died by Day 72 after tumor injection whereas 50% of the mice in the enzalutamide treated groups survived. The tumors in these long term surviving animals completely disappeared eight weeks after initiation of the drug treatment.

Immunohistochemistry study on formalin-fixed and paraffin-embedded (FFPE) mouse brain tumor tissue demonstrated that cell surface CSC marker CD133 and oncogene c-Myc expressions were both significantly decreased in enzalutamide-treated mice tumors compared with those in control group (**Figures 6E, F**). It was also interesting to observe that although the percentages of AR-positive cells in the tumor did not show significant differences between enzalutamide-treated and control groups, the AR expression levels or IHC staining intensity was decreased after enzalutamide treatment. Comparing with the control tissues without drug treatment showing AR staining mostly in the nuclei (95.4%), the tumor brain specimen after enzalutamide treatment showed a significantly higher percentage of cells with cytosol dominant distribution (96.7%) (p <0.05) (**Figure 6E**).

### All CD133+ Cells Are AR Positive *In Vivo* But AR Expression Is Not Specific for Glioma CSCs

Confocal microscopy was performed to study the expression patterns of AR and glioma CSC marker genes on FFPE mouse brain tumor specimens. AR in orthotopically growing tumor cells showed variable staining intensities (**Figure 7A**) and nuclear-dominant expression patterns but was also detectable in cytosol (**Figure 7B**). Clusters (thick arrow) as well as individually distributed (arrowheads) CD133+ CSCs were shown in **Figure 7A**. CD133 staining pattern was consistent with a cell membrane distribution (**Figure 7B**). Due to the different subcellular distribution patterns, co-localization rates of AR and CD133+ cell were manually counted. All CD133+ cells (100.0% ± 0.0%) were positively stained for AR. It is interesting for us to observe that individually localized CD133+ cells showed high rate of co-localization with cells with higher intensity of AR staining defined by >50% or maximal intensity (91.3% ± 7.5%) (arrowheads, **Figure 7A**). Clusters of CD133+ CSCs, as in the dash line box in **Figure 7A**, were all AR positive but not always in high AR intensity cells (**Figure 7B**). AR expression in CD133-negative (AR+/CD133−) cells was also seen but mostly (80.2% ± 8.3%) in cells with lower intensity of AR staining (thin arrows, **Figures 7A, B**). The staining pattern indicates that higher AR expression level is associated with cells with higher stemness. Similarly, All Nanog-expressing (Nanog+) cells were AR+ per manual counting. More impressively, the Nanog-weighted Nanog/AR co-localization coefficient equaled 0.75 based on software analyses which means 75% of positive Nanog staining signals co-localize with AR staining. Again we noticed that Nanog+ cells tend to have stronger AR stainings as well with nearly all Nanog+ cells (89.5% ± 10.1%) showing AR signal intensities above the 50% threshold of the maximum (arrowheads, **Figure 7C**). In contrast, a large portion of AR+ cells (76.7% ± 10.2%) showed no detectable Nanog staining (thin arrows, **Figure 7C**). Again these cells (AR+/Nanog−) mostly showed weak AR staining intensity (≤50% of maximum) (90.2% ± 5.1%), but still significantly higher than the background as shown in the negative control (**Figure 7D**). Data further supporting these observations were from the software-based co-localization analyses which showed that AR-weighted Nanog/AR co-localization coefficient was 0.09, in contrast to 0.75 for Nanog-weighted number. The results were interpreted as: although all Nanog+ cells were also AR+ and Nanog protein staining heavily co-localized with AR protein, AR expression was much more diffusely seen in tumor cells than Nanog due to the findings that many weakly AR+ cells were Nanog negative.

## Discussion

The presence of specific steroid hormone (estrogen, progesterone, or androgen)-binding receptors has been correlated with the clinical outcome and response to hormonal therapy in a number of different neoplasias, including breast, prostate and renal cell carcinoma (57). However, there is much less information available in brain tumors for steroid hormone receptor expressions or response to hormonal—particularly androgen—suppression. Previous studies, as well as our own, showed that androgen receptors were consistently detected in a higher proportion of gliomas (17, 27, 28, 31). These findings may help to explain the gender difference in GBM incidence and indicate that AR might be a promising therapeutic target for treating GBM.

Surprisingly, we have found a similar high proportion of GBM in female patients that expresses AR, and a GBM cell line derived from a female patient, Ln229, also responds to androgen receptor antagonists in a way similar to cell lines derived from male patients (**Figure 2**). The role of AR in gliomagenesis in female patients is worth further studying. It has been reported that following brain injury in rodent and bird models, astrocyte aromatase expression is upregulated transiently starting from hours post-injury and lasting for a few weeks (58–60). These data provide a possible mechanism for the upregulation of AR and/or secondary AR self-activation through aromatase-mediated testosterone conversion/depletion, which chronically could induce AR overexpression and/or become androgen independent. Ovary and adrenal glands produce dehydroepiandrosterone (DHEA), androstenedione and testosterone. Furthermore, for postmenopausal women, the common age for GBM diagnosis, the ovary becomes an androgen-secreting organ (61). Androgen antagonists thus may play an equally therapeutic role in both genders.

We have observed for the first time very consistent results from our studies both *in vitro* and *in vivo* that AR blockade downregulates the expression levels of majority of the tested GBM CSC-specific marker genes. Cell culture studies showed significant reductions of cancer stem cell genes at both mRNA and protein levels in both human and mouse cell lines cultured anchorage dependently as well as in tumor spheroids. AR genes are very conservative between human and mouse. AR antagonists significantly suppress the expression of c-Myc, whose activity is required for proliferation, growth, and survival of glioma CSCs (56). The results strongly suggest that AR may be involved in the process of gliomagenesis and act as an essential factor for glioma CSC maintenance and/or proliferation, which is consistent with the findings that androgen/AR promotes neural stem cell proliferation (62). We acknowledge that the functions of androgen/AR in embryonic and somatic stem cells have been shown to be tissue type-dependent, and the role of AR in cancer stem cells have been controversial which again demonstrates the significance of our studies (63, 64). Supporting evidence for our hypothesis further stems from observations that AR expression is induced in the glial cells in animal brains after injuries (excitotoxic injury or stab wound induced) in both male and female rat and avian models (65, 66). Although there is some discrepancy on whether reactive astrocytes or microglial cells are the source of an overexpression of AR, the data do suggest that AR might be playing a role in pathological conditions such as carcinogenesis in glial cells. In the rat model, AR was seen to be expressed at low levels in some cortex and hippocampal neurons but not in non-stimulated astrocytes, and no overexpression was seen in neurons adjacent to injury site. In mouse GBM specimens, we found negative staining of AR in adjacent and contralateral normal brain tissue. It is interesting to note that in patients with epilepsy, their temporal lobectomy tissue also showed some degree of elevation of AR expression when compared to brain tissues from autopsy of normal brain which again suggests the possible involvement of AR in the pathologic process of brain (**Figure 1**).

It is still debatable which CSC marker genes currently studied represent true stemness in these precursor tumor cells. Thus we have combined both *in vitro* and *in vivo* studies including tumor spheroid formation assays, limiting dilution assays *in vitro* and *in vivo*, CSC marker studies from TCGA database and from our RNAseq studies as well as IHC and confocal microscopy on multiple CSC marker genes to confirm that AR is essential for maintenance of glioma CSC population. Our results are consistent with other studies demonstrating that AR can bind directly to Nanog gene promotor and promote cancer cell stemness in hepatocellular carcinoma and ovarian cancer (67, 68). Although our *in vivo* tumor model demonstrated a high percentage of CSCs in mouse GBM by IHC and confocal microscopy, there are very low abundance of CD133+ cells when GBM cells were cultured *in vitro*. Our findings are consistent with what have been reported previously that *in vitro* cultured GBM cells contain a very low percentage (0.3–5%) of CD133+ cells particularly in high serum conditions which induce differentiated state of the tumor cells that are CD133− (69–72). Meanwhile, Jensen et al. reported that, when they implanted the U87MG cells into the mouse brain, they found that there was 30–40% CD133+ cells in the mouse GBM tissue developed, significantly higher than that in *in vitro* conditions. Interestingly, CD133+ cells also exhibited clustered distribution pattern in the culture tumor spheroids and brain tumor tissue as seen in our studies (**Figure 6E**) (73). However, AR antagonists, both enzalutamide and bicalutamide, demonstrated significant efficacy in suppressing cell proliferation after only two days of treatment in cultured GBM cell lines indicating that AR may not only promote CSCs but also cell proliferation. Indeed, our unpublished studies also showed that blocking AR could induce G2/M cell cycling arrest in GBM cell lines. AR blockade can significantly downregulate c-Myc protein levels in GBM cells both *in vitro* and *in vivo* (**Figures 2** and **6**) with known cellular functions of c-Myc in cell proliferation and glycolysis in glioblastomas (74, 75). Gene ontology from RNA-seq results also confirmed the additional functions of AR in cell cycling/proliferation particularly by regulating Hippo, PI3K/Akt and MAPK signaling pathways which can all contribute to both CSC and differentiated cancer cell divisions (**Figure 5**). Our results from confocal microscopy also confirmed that AR expression can be detected in both CD133+ and CD133− tumor cells although AR expression levels, as indicated by staining intensity, were shown to be highest in isolated CD133+ cells than clustered CD133+ cells and AR+/CD133− cell (**Figures 7A, B**). Similar results were found using Nanog as another CSC marker (**Figure 7C**). Questions still remain whether the higher AR staining intensity is due to higher protein expression level or protein aggregation in cells. Based on these results, we hypothesize that glioma CSCs may be more dependent on AR expression/functions for maintenance and/or survival than more differentiated tumor cells. Cancer stem cells (CSCs), although composing only a small portion of the tumor cell population, have the highest AR expression levels/staining intensities (AR+++) which decrease as the CSCs differentiate into partially differentiated cancer stem cells (PDCCs) (AR++) and subsequently into differentiated cancer cells (DCCs) with the lowest AR expression level/intensity (AR+) (**Figure 7E**).

We are currently conducting further experiments including overexpression of c-Myc in GBM cell lines to potentially overcome the effects from AR suppression, as well as CRISPR/CAS9-mediated AR knockout in GBM cell lines to investigate the multi-faceted functions of AR in GBM tumor growth. Nonetheless, the particular efficacy of AR blockade in suppressing glioma CSCs signify the importance of further research on this novel target with its potential to overcome the tumor resistance mediated by CSCs to current standard care with RT and/or chemotherapy.

AR antagonists have been used to treat prostate cancer for more than 35 years with extensive clinical experience and accumulation of biological data (76). Enzalutamide, a new generation of AR blockade drug that is FDA-approved for metastatic prostate cancer, which also demonstrated excellent brain penetration capability, provides us a readily testable drug for repurposing in GBM patients (77). Enzalutamide, unlike the previous generations of AR antagonist drugs such as bicalutamide and flutamide, not only can prevent androgen and AR binding, but also block the nuclear import of AR including some of the AR splicing variants (AR-Vs). AR-Vs have been reported to contribute to prostate cancer progression through induction of epithelial-to-mesenchymal transition and acquisition of stem cell characteristics (78). The expression of AR-Vs lacking the c-terminal ligand-binding domain (LBD) was found to be increased in androgen-independent and metastatic prostate cancers (79, 80). Some of these AR-Vs such as AR-V7, are constitutively active and localized in the nuclear compartment, and their transcriptional activity is not regulated by androgens. However, Zhan et al. (81) also reported that, when expressed alone in cells, some AR-Vs (e.g., AR-V1, AR-V4, and AR-V6) localize mainly in the cytoplasm but can dimerize with AR-V7 or widetype AR to be nuclear localized. There is very limited information on whether AR-Vs are present in GBM except for a preliminary study from Zalcman et al., indicating ~30% of the glioblastomas in patients expressed a constitutively active AR-splice-variant (AR-V7/AR3) lacking the LBD (30). Whether AR-V7 is expressed in U87MG cells are controversial (30, 82, 83). No information exists whether other types of AR-Vs present in GBM although data from Zalcman et al. strongly indicate the presence of castration-resistance of the tumor from the very beginning of pathogenesis of GBM which is different from prostate cancer that usually becomes so after prolonged androgen deprivation therapy. If that is the case, enzalutamide and other newly generation of AR antagonists such as apalutamide could provide superior GBM control benefit compared to older generation drugs or antagonists/agonists of gonadotropin-releasing hormone (GnRH) that suppress androgen production, as having been demonstrated by a phase III clinical trial on metastatic prostate cancer (84). Our results showed that, when cultured *in vitro*, GBM cells including the mouse MGPP3 cells had cytosol dominant distribution pattern of AR (**Figure 2**) with or without enzalutamide treatment. However, IHC staining on both mouse and human brain tumor specimens demonstrated nearly 100% nuclear localization of AR but more cytosol dominant distribution pattern after enzalutamide treatment *in vivo*. These results, although cannot conclude whether there are cytosol-located AR-Vs in these GBM cells, do indicate that the testosterone concentrations in culture medium might not be high enough as in brain tissues to induce translocation of AR to nucleus when cultured *in vitro*. Our results also provided evidence that enzalutamide can successfully block the AR translocation to the nucleus as reported before (85).

With our results in GBM and previous studies from prostate cancer showing specific cancer stem cell suppression, AR antagonists could be good therapeutic candidates and repurposed in the treatment of GBM, particularly when combined with current standard care modalities such as temozolomide and/or radiation therapy, which cancer stem cells are known to be resistant to (3, 86, 87). We also acknowledge that *in vitro* effective dose of enzalutamide in GBM cells (IC50: ~40 µM) or for spheroids (60–120 µM) from our study seems to be higher than the therapeutic dose in plasma achievable *in vivo*. The phase I/II study revealed that the minimum (predose) plasma concentrations (C_min_) at steady state in the 150 mg PO daily dose cohort of patients for enzalutamide is about 20 µM although nearly doubling of the concentration can be achieved if using maximal toxicity dose (88). Preclinical studies on prostate cancer cells demonstrated Ki (inhibition constant) of enzalutamide is 86 nM and the IC50 of enzalutamide to suppress widetype AR activation by testosterone based on reporter gene transcription assays is 219 nM. The IC50 of enzalutamide in cell viability assays for VcaP, an AR dependent prostate cancer cell line, is 410 nM (89). These numbers are drastically lower than the IC50s of cell proliferation assays we observed in GBM cell lines. One explanation of the difference is the difference in experimental conditions. For example, the culturing time after adding the drug prior to cell proliferation assays was much longer than ours (4 days *vs.* 2 days). The reason we chose 2 days culturing time after adding the drug instead of 4 days is that after 3 days, we started to see synchronized cell apoptosis in U87MG and other GBM cell lines such as U138MG which develops rapidly (data not shown). It is noted that in the studies by Zalcman et al., the concentrations of enzalutamide used to treat GBM cells for 48 h, same as what we did, were from 10 to 80 µM, consistent to our data (30). We also would like to point out that the IC50s reported by Moilanen et al. was measured under testosterone stimulation (mibolerone) which very likely would have caused left-shift of the survival curve. However, Xue et al. reported that the IC50s of enzalutamide on different prostate cell lines such as LnCap, C4-2B, 22Rv1 and VCaP were 42, 20, 36, and 30 µM without testosterone stimulation (90). Although the authors did not specify the culturing time for each cell line after adding the drug but stated the shortest culturing time is 72 h. These IC50s are very similar to our results from GBM cell lines and probably reflecting the conditions *in vivo* better with the testosterone levels in elderly patients, male or female, being very low around the average ages of GBM diagnosis. Another explanation on higher IC50s seen in GBM cell lines is the potential presence of AR-Vs, as discussed above that may render GBM cells much more resistant to AR antagonists comparing to androgen-dependent prostate cancer cells, which will keep us in mind when developing future clinical trials repurposing AR antagonists for GBM treatment. Despite of these explanations, enzalutamide in this range of concentrations (40–80 µM) might involve a non-canonical target(s) with off-target effects which warrants further studies. Arguing against this hypothesis are the published data showing that knocking down of AR by siRNA resulted in significant inhibitory effects on GBM cell growth *in vitro* although whether the effects were mainly on differentiated tumor cells or CSCs is unclear (30).

Nevertheless, our results support the potential of repurposing AR antagonists for GBM treatment. Enzalutamide showed significant efficacy in the syngeneic orthotopic mouse GBM model, although well tolerated, only 50% of the mice survived long term (**Figure 6**). We did observe significantly more weight gain in drug-treated mice which is a well-known side effect of androgen deprivation therapy. It is also noted that the survival curve after enzalutamide treatment showed initially the same pattern as control group but separated eventually indicating a heterogeneity of tumor response to the drug. Our pre-clinical results indicate that likely further dose escalation study for GBM patients or combining this drug with other standard care modalities for GBM such as temozolomide and/or RT may be necessary to further improve the outcome, as supported by the most recently published data from Werner et al. in the flank implant tumor model (31).

In summary, our data demonstrated tumor suppressive effects of AR antagonists, particularly enzalutamide, in GBM cell lines and, for the first time, in an orthotopic mouse model. Potential mechanism of the drug effects appear to be at least partly mediated through inhibition of cancer stem cell *via* AR in gliomagenesis and may provide us with a novel target for GBM treatment.

## Data Availability Statement

The original contributions presented in the study are publicly available. This data can be found here: https://www.ncbi.nlm.nih.gov/geo/query/acc.cgi?acc=GSE174295.

## Ethics Statement

The animal study was reviewed and approved by Institutional Animal Care and Use Committee of the University of Nebraska Medical Center.

## Author Contributions

CZ (16th author) and NZ designed the experiments and wrote the manuscript. NZ, CZ (16th author), FW, SA, KL, CZ (5th author), SC, DD, MP, BG, PZ, and SW did the experiments and analyzed the data. SB, TB, CL, and TH interpreted the data and revised the manuscript. All authors contributed to the article and approved the submitted version.

## Funding

The work was supported by the National Institute of General Medical Sciences (1U54GM115458-01).

## Conflict of Interest

The authors declare that the research was conducted in the absence of any commercial or financial relationships that could be construed as a potential conflict of interest.

## Supplementary Material

Supplementary Figure 1 Western blotting results demonstrate positive AR expression at different levels in GBM cell lines of U87MG, U138MG, Ln229, MGPP3, M059K, and A172. Prostate cell line LnCap is used as a positive control.

Supplementary Figure 2 Western blotting results of FOXO3a, GATA4, CDC25A, GADD45A, and beta-actin of cultured U87MG and MGPP3 cells after the treatment of enzalutamide (80 µM) at different time points. 8h, 24h, 48h: cells were treated with the drug for 8, 24 or 48 hours. All experiments were performed with three independent replicates.
