# Supplementary figures and images for: Androgen Receptor, Although Not a Specific Marker For, Is a Novel Target to Suppress Glioma Stem Cells as a Therapeutic Strategy for Glioblastoma
Source: Front Oncol. 2021 May 21;11:616625. doi: 10.3389/fonc.2021.616625 (PMC8175980; doi:10.3389/fonc.2021.616625)

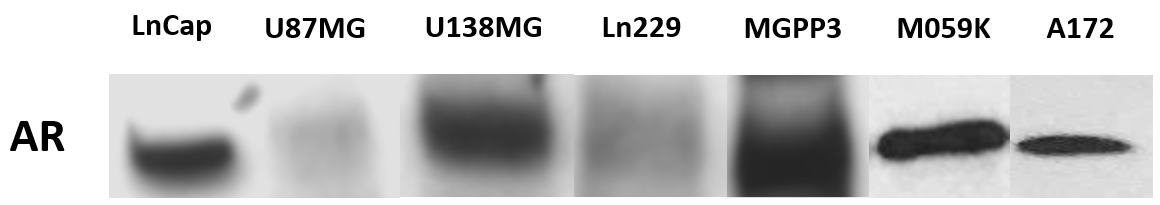

Supplement: Supplementary Figure 1 — Western blotting results demonstrate positive AR expression at different levels in GBM cell lines of U87MG, U138MG, Ln229, MGPP3, M059K, and A172. Prostate cell line LnCap is used as a positive control. [file Image_1.tif]

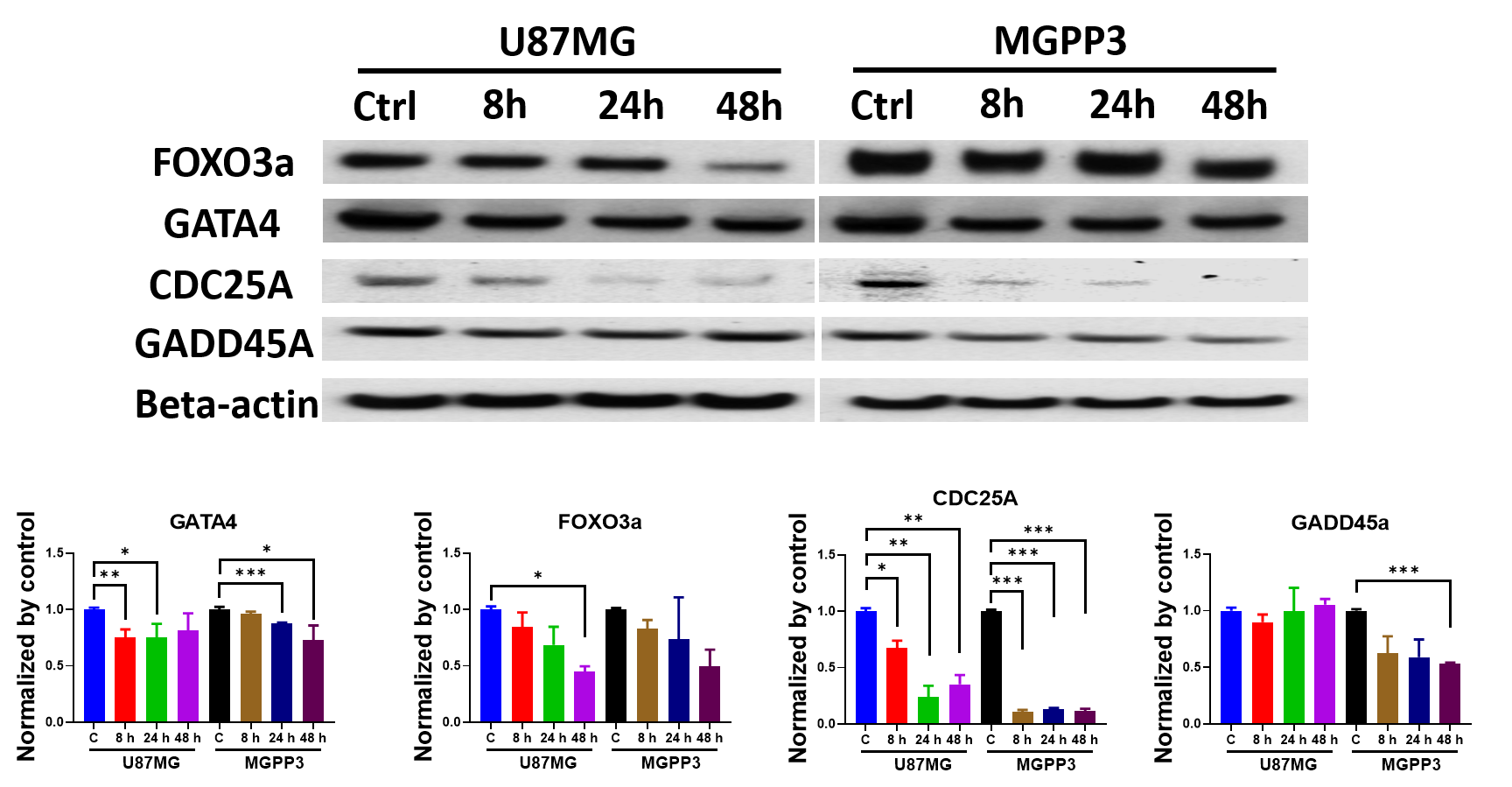

Supplement: Supplementary Figure 2 — Western blotting results of FOXO3a, GATA4, CDC25A, GADD45A, and beta-actin of cultured U87MG and MGPP3 cells after the treatment of enzalutamide (80 µM) at different time points. 8h, 24h, 48h: cells were treated with the drug for 8, 24 or 48 hours. All experiments were performed with three independent replicates. [file Image_2.tif]
